# In Vitro Research Methods Used to Evaluate Shaping Ability of Rotary Endodontic Files—A Literature Review

**DOI:** 10.3390/dj12100334

**Published:** 2024-10-21

**Authors:** Ranya F. Elemam, Ana Mano Azul, João Dias, Khaled El Sahli, Renato de Toledo Leonardo

**Affiliations:** 1 Restorative Dental Science Department, College of Dentistry, Gulf Medical University, Ajman P.O. Box 4184, United Arab Emirates; 2 Egas Moniz School of Health and Science, Monte da Caparica, 2829-511 Almada, Portugal; 3 Egas Moniz Center for Interdisciplinary Research, Monte de Caparica, 2829-511 Almada, Portugal; 4 The Libyan Authority for Scientific Research, Tripoli P.O. Box 80045, Libya; 5 Department of Restorative Dentistry, School of Dentistry, São Paulo State University (Unesp), Araraquara 14801-903, SP, Brazil

**Keywords:** root canal preparation, canal transportation, centering ability, shaping ability, nickel–titanium, rotary

## Abstract

Background/Objectives: In this article, we present a literature review of methods used to measure the shaping ability of endodontic rotary files, including the selection of endodontic sample type (extracted teeth versus simulated blocks) and an imaging evaluation method. This review was conducted as background research to identify concerns that arise when designing research studies in this domain and propose how the field can plan more systematic studies going forward. Methods: A literature search was conducted using PubMed, MEDLINE, Embase, ScienceDirect, Scopus, and e B-on databases, including studies published in English from January 2010 to June 2024. Only studies that specified in vitro or ex vivo methods for evaluating the endodontic performance of NiTi rotary files on canal transportation and centering ability were considered. Results: A total of 86 studies met the inclusion criteria from an initial pool of 651. Of these, 67 studies used extracted teeth, while 20 utilized simulated root canals in resin blocks. For evaluation methods, 55 studies employed Micro-Computed Tomography and Cone-Beam Computed Tomography (MCT + CBCT), 30 used Double Digital Images/Radiographs/Photographs (DDIR + DDIP) with software analysis, 1 used both DDIR and MCT, 1 used high-precision nano-CT, and 1 used a digital single-lens reflex (DSLR) camera. Conclusions: The findings indicate that the MCT method and its advanced variations appear superior in many cases for evaluating the quality of root canal instrumentation due to their ability to provide detailed three-dimensional images. We also discuss the pros and cons of other evaluation methods, including CBCT and DDIR. Finally, we identify important factors to consider for optimizing future cross-study comparisons. This work highlights the importance of being familiar with shaping ability assessment methods as new instruments are introduced to the market.

## 1. Introduction

NiTi rotary instruments show significant improvement in the technical quality of endodontic therapy due to their unique flexibility and time-saving properties [1]. In addition, the wide range of their designs and cross-sectional patterns have led to many experimental studies being performed by scientists to evaluate their clinical performance, mainly by studying the instruments’ cleaning ability, shaping ability, and safety concerns [2]. The shaping ability of endodontic nickel–titanium rotary instruments is still a major concern of endodontics researchers. It indicates the potential of the endodontic files to shape the root canal—specially curved canals—without causing any aberrations. This is attained by assessing whether the file is straightening the curvature of the canal, its ability to be centered, and its ability to maintain the centering of the canal with minimal transportation.

Schilder [3] proposed clear design objectives when operators are shaping the root canal; when the objectives are followed, cleaning will be easily facilitated, and obturation will produce an optimal seal. These objective areas, briefly, are as follows:

Taper—A continuously tapered preparation shape should be formed.

Canal axis—The position of the canal axis should be sustained in the center of the root with no deviation.

Foramen—The original position of the foramen should be maintained, and it should not be enlarged.

The shaping ability of endodontic files should reflect those clear objectives to obtain an ideal-shaped canal, with a preparation that involves negligible canal transportation with optimally centered preparations [4].

There are many evaluation parameters used in the literature to investigate the shaping ability of instruments. They include the change in the root canal cross-sectional area, degree of canal transportation, centering ability, minimum remaining dentine thickness in the mesial and furcal directions, taper and flow of “the prepared root”, smoothening of the canal walls, change in curvature angulation, centering ratio, working time, fracture of instruments, canal aberrations, and working length [5]. However, transportation and centering ability are used most frequently in the majority of studies; as a result, these factors were considered in this review.

Canal Transportation is defined as the removal of canal wall structure on the outside curve in the apical half of the canal due to the tendency of files to restore them to their original linear shape during canal preparation; this may lead to ledge formation and possible perforation [6]. Centering ability is the ability to keep the instruments centered to provide an accurate enlargement without excessive weakening of the root structure [7]. The canal-centering ratio is the difference between the instrumented and non-instrumented canals, which measures the ability of an instrument to stay centered [8].

Numerous devices and methods are used in the shaping ability evaluation process, which includes silicon impression, muffle system, and radiograph superimposition techniques. These techniques are effectively documented in endodontic research. Yet, limitations are well-recognized, encouraging a search for new evaluation methods with advanced capacities that permit both quantitative and qualitative three-dimensional (3D) assessments of the root canal [9]. Such evaluations are important to clinicians and researchers because their consideration is valued in the selection of a particular rotary NiTi instrument for clinical practice [10].

The purpose of this review article is to investigate and analyze the literature that examined the shaping ability of endodontic rotary files to weigh the pros and cons of different methods used for this assessment based on canal transportation and centering ability parameters. Importantly, this review covers in vitro or ex vivo samples only, and thus, the search was conducted to explore the research methods literature rather than to test a clinical question or hypothesis. The review was conducted to identify the issues and challenges that come into play when designing studies evaluating rotary files and was not designed to be a systematic clinical review to objectively compare methods or file performance. In particular, we focused on the heterogeneity of endodontic sample type in the literature (extracted tooth versus simulated tooth, as well as which type of tooth) to identify that this presents challenges in making objective comparisons of evaluation methods. This approach was intended to guide future study design and harmonization of clinical evaluations.

## 2. Materials and Methods

The first author of this paper reviewed the literature for relevant published studies on methods used in the evaluation of the shaping ability of rotary files in the context of endodontics. Six databases were searched: PubMed, MEDLINE, Embase, ScienceDirect, Scopus, and e B-on database. In the initial search phase, studies were identified using the following pair of search term strings in each database: “evaluating shaping ability of NiTi rotary endodontic files canal transportation” and “evaluating shaping ability of NiTi rotary endodontic files centering ability”. The First Author included temporal filters (publication date) in each search for the years covered (2010–2024). Upon collection, they were fully reviewed to ensure that they met the inclusion/exclusion criteria.

Inclusion criteria were in vitro and ex vivo studies that described, in the abstract and/or Materials and Methods section, use of either extracted teeth or simulated teeth and evaluation method to evaluate endodontic rotary file performance on the following parameters: transportation or centering ability parameters and evaluating preparation quality based on shaping ability of rotary files. Searches were limited to studies written in English with human-extracted teeth or experimental blocks and published between January 2010 and June 2024. The decision to begin the search in 2010 was based on this study being conducted as a literature search for a Master’s thesis that used a 10-year retroactive literature search approach. For the current publication, we updated references through the gap between the initial thesis and submission for publication.

The exclusion criteria consisted of studies that failed to meet the inclusion criteria. If a study did not define transportation/centering ability as parameters or report shaping ability to evaluate rotary files, it was excluded. All systematic reviews, literature reviews, case series, study reports, and studies that expressed opinions were read but not included in the analysis since they did not contribute new data points on the topic of this review, which was asking the question, which methods are used in vitro/ex vivo to evaluate rotary file performance? Studies that allocated cleaning rather than shaping and/or dealing with the identification of bacterial species, material science, or clinical settings were also excluded because they did not evaluate the topic of this review. Lastly, studies in which publication keywords did not match the subject of the search, as well as non-English language studies, were excluded as they did not evaluate the topic of this review.

Because this was part of an independent Master’s thesis project, only the student conducting the thesis and screened the initial pool of studies identified in the search. Information from included studies was tabulated as shown in Table 1 and Table 2. The First author then worked in collaboration with the other authors to validate, analyze, review and edit, visualize, and administer the project.

## 3. Results

**Figure 1:**
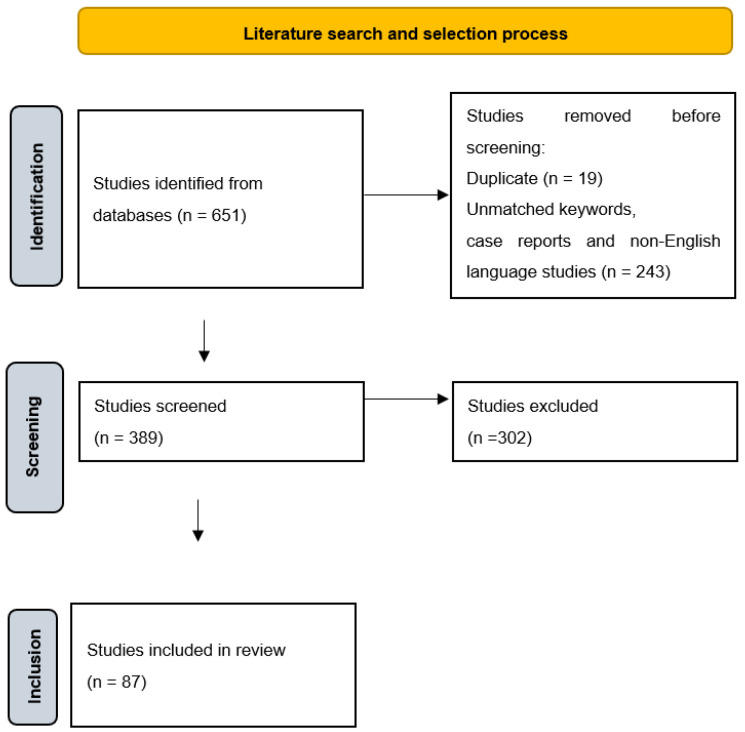
Literature search and selection process.

The search of the selected databases yielded 651 studies. After the exclusion criteria were applied, duplicates across databases, case reports, and non-English language studies were excluded. After these exclusions, 389 published studies relevant to the instrumentation of root canals were identified. Titles and abstracts were additionally evaluated, and full texts of the selected studies were then obtained. Articles were further evaluated through a detailed reading of the methods to confirm their inclusion. After these screening procedures, only 87 were kept, as shown in Figure 1.

From the selected studies, the following data were extracted from the Abstract and Materials and Methods sections: endodontic sample type, methods used to evaluate transportation or centering ability (Micro-Computed Tomography (MCT)-NRecon v.1.6.4; Bruker micro-CT, Cone-Beam Computed Tomography (CBCT) -KaVo OP 3D Vision (Kavo Dental, Biberach, Germany), Digital Single-Lens Reflex Camera (version 1.0 (build 1.0.10.7462), × 64 Edition, copyright 2004–2017 Cybermed, Korea and license key 670,094,709.), High-Precision Nano-Computed Tomography (nano-CT), (ver. 2.1.0.2, SkyScan, Kontich, Belgium), Double Digital Images Photographs (DDIP) with either Adobe Photoshop (Adobe Photoshop CS6, Adobe Systems Inc. San Jose. CA. USA), Fiji ImageJ software v.1.49n, Image-Pro Plus software Image-Pro Plus 6.0 (Media Cybernetics, Warrendale, PA, USA), or AutoCAD software (version 23.0, 2018), Double Digital Image Radiographs (DDIR) with either MCT (Skyscan 1172; SkyScan b.v.b.a, Aartselaar, Belgium), Adobe Photoshop CS6 version 13.0 (Adobe Systems, San Jose, CA) or AutoCAD software 2006 and 2008 software (Autodesk Inc., San Francisco, CA, USA), as shown in Table 1.

The distribution of studies according to the types of samples, the evaluation methods used, and the evaluated parameters are shown in Table 2.

Table 2 reveals that mandibular molars and simulated block types of samples constituted about 85% of the selected studies. For mandibular molars, more than 92% of the studies used Micro-Computed Tomography (MCT), Cone-Beam Computed Tomography (CBCT), or Double Digital Image Radiographs (DDIR) with the AutoCAD software. For simulated blocks, about 77% of the studies used Double Digital Image Photographs (DDIP) with either Adobe Photoshop or AutoCAD software.

**Figure 2:**
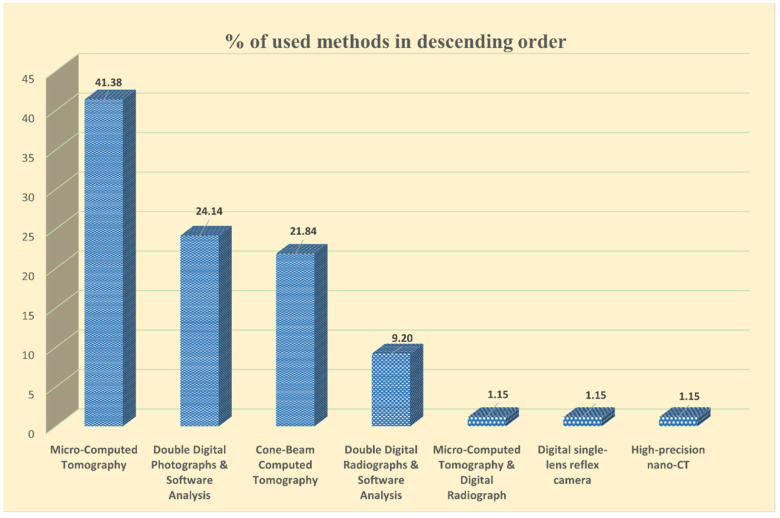
Percentage of evaluation methods used in descending order.

Reviewing all the selected study articles in detail resulted in the ranking of the evaluation methods used to assess shaping ability in terms of their frequency of use across all sample types, as shown in Figure 2.

## 4. Discussion

### 4.1. Types of Endodontic Samples

A strong majority (77%) of studies on post-operative root canal shape or changes in root canal morphology were performed in extracted teeth rather than in simulated samples. Molar teeth were the most selected type, and the highest percentage was found on mandibular molars [9,14,16,18,20,21,22,24,25,26,27,32,33,34,35,38,39,43,46,47,48,50,52,53,55,58,63,65,67,68,72,74,76,79,80,82,85,86,88,90,92,93,95,96,97]. Few experiments were performed on maxillary molars [13,15,40,42,54,60,71,77], and only three studies were performed on both maxillary and mandibular molars [22,31,56].

Researchers were interested in evaluating the quality of shaping ability of endodontic files in molar teeth because these teeth are the most treated within the general dental practice [34,98,99]. The mesial root was the preferred root for this type of experiment, usually because they are curved, with the greatest curvature in the mesio-buccal canal. This anatomical feature of the curved mesio-buccal canals often induces a greater challenge [32,100] and generates greater canal transportation by instrumentation than most other root canals [76].

Studies that used simulated canals in resin blocks were few [10,17,28,30,45,49,51,57,59,62,66,69,70,75,81,83,89,91,94,101]; in the cases they were chosen, it was due to these samples being reliable, valid, and credited models for testing canal preparation techniques and instrument ability [16,18]. One study used simulated blocks in the shape of molars [51]; this artificial molar tooth model is made of a material that is closely equal to natural dentin so that each step of the treatment is comparable to real clinical practice. The studies that used resin blocks confirmed that those blocks can give better standardization and are able to reduce the variability that exists in the human root canal anatomy [17,19,28,30,66,69,70,81,89,94], providing strictly controlled laboratory conditions [83]. They also allow a direct comparison of the shapes obtained with different movements [91] and with different instruments [70,81]. However, those simulated canals in resin block models may neither match the various anatomical configurations in actual tooth structure nor match the clinical setup; the patient factor for this clinical outcome might not be considered [70].

### 4.2. Main Evaluation Methods

Three main methods were cited in our reviewed literature to evaluate the performance of root canal instrumentation. These are Double Digital Images, MCT, and CBCT.

#### 4.2.1. Double Digital Images Evaluation Method

The Double Digital Images or Standardized Images technique has traditionally been one of the most used methods in endodontic research studies and was widely mentioned in this review [9,10,19,23,29,45,49,51,56,57,58,59,60,62,66,69,70,73,75,78,80,81,83,84,86,89,90,92,93,94].

This technique allows a direct analysis of post-instrumentation changes in the root canal system and evaluates the tendency of instruments to maintain the original canal anatomy under standardized conditions in a simple approach [78,102]. The assessment of anatomic parameters like transportation, centering ability, and centering ratio was easily achieved when this technique was selected [19,23,29,102]. In addition, residual dentin and cutting efficiency of different instruments could be evaluated [103,104,105].

The Double Digital Radiographs/Photographs Images (DDIR/DDIP) method was named double because of the double time exposure—one before and one after instrumentation. It is also called standardized because the technique has to maintain the same image exposure position each time [106]. It is relatively simple to perform, starting by digitizing the radiographs or photographs so that the operators would have the advantage of controlling contrast and brightness [107], then superimposing post- and pre-instrumentation images using computer software to evaluate the degree of canal transportation or other parameters.

When the Double Digital Images method uses the muffle system, it is called the Bramante technique or a modification of the muffle block technique [108], where a plaster block is placed around a resin or indexed experimental tooth [108]. The block can be custom machined and sectioned in various planes to allow exact repositioning of the complete block or sectioned parts of the tooth in the same position [109]. In our review, one study applied the Bramante technique [73] due to its low cost, simplicity, and adequacy, and it was considered sufficient for the assessment of the quality of root canal preparation [73].

Photographs and radiographs cannot be observed in a cross-sectional view [110]. All images received from this method are two-dimensional (2D) views. Deolivera et al. [46] defined the two dimensions as area and perimeter and the three dimensions as volume, surface area, and structure model index. In clinical radiographs, the 2D images are the clinical (mesiodistal) and proximal (buccolingual), which did not display the real transportation because teeth do not always show their maximum curvatures in the mesiodistal or buccolingual planes [111,112]. Accordingly, different adjustments were suggested to overcome this by implementing some modifications. A recommendation to take another angulated radiograph, commonly perpendicular to the first one, to provide an understanding of the third dimension was proposed; however, this still drops short of generating 3D data for quantitative analysis [113]. Another suggestion was to inspect the tooth, locate the position of maximum curvature, and set it perpendicular to the X-ray beam [114]. This modification was first suggested by Maggiore [114], allowing an exact evaluation of the angle and radius of the curvature [104]; however, it is still not indicated in cases of root canals with double curvatures because maximum curvatures in these canals normally occur in multiple planes [104].

Comparing DDIR to MCT for evaluating canal transportation showed similar statistical results. Although this outcome lacked clinical relevance, radiography is still a reliable and precise tool [60].

Double Digital Image Radiography (DDIR) illustrates a nondestructive approach, demonstrating slow exposure to radiation [110], ease of use, and low cost compared to MCT, and is preferable to the investigators [9]. All images were taken in two perpendicular directions, providing 2D estimates of 3D structures. This does not give an adequate and complete description of an object, leading to reduced accuracy in quantitative studies [89]. Interpretation of radiographs and images remains subjective [115] and lacks the ability to reveal volumetric information of the 3D view [89], making CT superior [9].

#### 4.2.2. MCT (Micro-Computed Tomography) Evaluation Method

MCT was the most-selected evaluation method within this review [14,18,20,24,26,27,32,33,34,35,37,38,39,40,41,42,43,46,47,48,53,54,55,60,61,63,64,65,67,68,71,76,77,82,87,88,89]. Authors specified the reason for this selection as the tool’s advantages, mainly the ability to obtain a 3D assessment of the root canal preparation [14,33,37,40,41,47,68,82]. One study used this method to anatomically match the sample to generate a calibration by having a reliable baseline and ensuring comparability of the groups by standardization [68]. However, Stern et al. chose this method for its accurate images, owing to the higher spatial resolution compared to conventional clinical scanners [87] and for the ability to overcome previous technique limitations [82].

MCT was described as a state-of-the-art method for examining the internal anatomy of teeth [102,116]. MCT can investigate root canal geometry based on a wide range of parameters, including apical transportation, centering ratio, volume changes, cross-sectional shape, taper, and anatomical structure of the root canal before and after instrumentation [2,18,20,24,26,27,95,117]. Its 3D capability works by collecting the 2D projections of X-rays through a specimen, which are then used to reconstruct a 3D image [118]. It has been shown that an initial scan that was used for comparison after the shaping procedure was enough to test the volume change of the canal [119].

Other advantages of the MCT method include its ability to detect anatomical complexities, such as accessory canals [120], C-shaped canals, and isthmuses [121,122]. MCT has emerged as a powerful tool for ex-vivo evaluation of root canal morphology due to its accuracy, noninvasive procedure [33,95], and 3D performance at both apical levels and point of maximum curvature [71].

The images provided through this method are induced at a resolution of 11.84 mm, proving to be an excellent method for the precise evaluation of the apical millimeters of instrumented root canals [90]. All transferred errors encountered by using radiographic or photographic methods are avoided [123]. This ability to image a very small structure made using MCT within this context is very demanding due to its higher magnification and significantly higher resolution compared to conventional tomography [119]. MCT has higher resolution due to lower voxel size. The importance of the resolution and quality of the image in scientific research outweigh the time required for the analysis [119].

Previous literature that used MCT analysis was hindered either by insufficient resolution [102] or projection errors [124]. Modern machines now offer better resolution and more accurate measurement software with the capability of matching multi-dimensional data from specimens before and after preparation [125]. For these reasons, the current generation of MCT is considered a superior method for evaluating the quality of root canal preparation techniques [126]. In spite of its high cost in requiring a well-trained operator and long scanning and reconstruction time [119], MCT is becoming a substantial educational tool for preclinical teaching in endodontics [127]. As technology continues to develop even further, higher resolution methods, such as nano-CT used in one recent study, may overtake MCT. Nano-CT currently has a resolution of <400 nanometers [128] and can image features at the cellular level, in addition to more macroscopic structural levels [129].

#### 4.2.3. CBCT (Cone-Beam Computed Tomography) Evaluation Method

CBCT is an extra-oral imaging method able to produce 3D scans of the orofacial skeleton [130]. This technique was selected by a number of studies in our review [11,15,16,17,21,22,25,28,30,31,36,44,46,50,52,72,74,79,85,95] along with its advanced type, including spiral [95] and ICAT [79,85]. One study used CBCT only for the sample selection process [46]. The authors declared that CBCT enabled the collection of homogeneous and balanced experimental groups to analyze 2D and 3D values of the sample to precisely interpret endodontic instrument behavior during root canal preparation.

The rationale behind CBCT selection as an evaluation method includes having noninvasive tool characteristics [36,85], an accurate reproduction of 3D evaluation [44,46,74,85,95,130], and the ability to detect alterations in canal curvature, dentin thickness, and root canal volume accurately [36,44,85,95,102,131,132].

CBCT could overcome the limitations of conventional radiography [133], such as compression of a 3D object into a 2D image, image distortion, and anatomic superimposition [73]. These are the main advantages of the CBCT method [134], in addition to the fast data acquisition of CBCT when compared to MCT [50,135]. It is used in clinical endodontic practice and more frequently in endodontic research to evaluate root canal morphology, fractures, and changes in prepared root canal [136] volume change, surface area, 3D root canal axis, thickness, surface convexity, and structure model index [32].

CBCT produces pure, clear images with the ability to record all the anatomic details of the teeth [32]; however, it has lower resolution compared to MCT, which may cause problems when enhancing data during imaging for research purposes [137].

The method name is due to the X-ray beam shape and the area the detector captures is a cylinder-shaped volume of data in one gain [138]. This makes CBCT very convenient both clinically and in the research lab [50], whilst MCT is better recommended for laboratory research only [119]. The main shortcomings of using the CBCT method are reduced sensitivity to minor anatomical changes, possibly due to reduced resolution compared to MCT [139]. Voxel size in MCT ranges from 19.6 μm to 39.2 μm isotropic voxel size compared with CBCT, which is larger [140,141]. The larger voxel size in CBCT imaging led to a partial volume effect, making it impossible to perform accurate measurements [142].

#### 4.2.4. Comparison of Evaluation Methods

This detailed discussion of the reviewed studies aimed to enhance understanding of the parameters to consider for selecting the most reliable method for evaluating the shaping ability of endodontic files, enabling the researchers to have a standard for testing the shaping ability of NiTi rotary instruments on a strong evidence base. This would further serve as a reference to clinical practitioners to promote better harmonization of tool assessment approaches across sites and studies.

The measurement of apical transportation can be particularly challenging because of the fact that there is no gold standard method for its assessment, as all methods chosen by researchers have limitations [113]. Additionally, apical transportation itself is difficult to measure because no standard exists for this measurement [9,84]. Lastly, we have determined that it is challenging to compare studies that assessed root canal transportation and centering ability due to a lack of standardized evaluation methods among the reviewed studies. Studies on canal shaping affected by instrumentation need to be homogeneous with respect to multiple factors such as canal shape and size, sample model nature, proper superimposition of before and after instrumentation images, the selected method, and the study design to objectively evaluate and compare the tools used for evaluation and achieve the optimal recommendation for any given evaluation.

In summary, the Double Digital Images (DDIR + DDIP) technique is a simple method offering 2D photographs of the sample, while MCT and CBCT present a 3D image. Both CT and CBCT are preferred due to their ability to capture images in three dimensions with accurate measurements, providing an opportunity for various slices of the same images. They also have a high efficiency in detecting anatomical complexities in the root canal system. They are both superior methods in evaluating and assessing canal preparation quality and could help in sample selection; however, they have a larger radiation exposure, longer time, and complex procedure compared with the Double Digital methods.

#### 4.2.5. Study Limitations

Although this study used specific search keywords and screened a dense body of literature, it was intended as a discussion of the pros and cons of different rotary file performance evaluation methods and not as a systematic review. Moreover, the review covered only in vitro/ex vivo methods assessment and was not intended to be used as a clinical tool for direct in vivo evaluation. Because this was a literature review rather than a systematic review, certain constraints in the study could be removed in future systematic reviews. For example, searches could be conducted spanning more objectively defined time periods (i.e., longer to explore history of methods or shorter to focus on the most modern technologies), and more combinations of keywords might be used to conduct a systematic review of this topic. In the current analysis, we excluded other review articles because they do not present original research data points, and the goal of the project was to focus on original research and data collection methods. However, future systematic reviews on this topic might make use of existing review articles for faster and more thorough identification of original research studies. Finally, we limited our search to articles written in English; future studies could remove this constraint to minimize any geographic bias that English-language-only studies may have imparted.

## 5. Conclusions

This review conducted searches and analyses on canal transportation and centering ability evaluation parameters. Here, 651 publications were identified and screened, resulting in 87 studies being selected and analyzed. Some conclusions and recommendations are the following:

Evaluating the shaping ability of root canal files becomes essential with the gradual introduction of new instruments to the market.

MCT is an outstanding method for evaluating transportation and centering ability, with the highest resolution and ability to assess in 3D. CBCT followed closely behind MCT in our literature review, with similar assessment performance to MCT but with lower resolution and lower ability to resolve small anatomical changes. The Double Digital Images (DDI) technique is also an excellent, low-cost, and simple method, particularly for evaluating whether canal anatomy remains the same after instrumentation use. However, photographs cannot be reconstructed in 3D, which reduces the ease of volumetric assessment and the ability to detect anatomic features that change out of the plane of measurement.

Future studies evaluating rotary file performance should be based on the use of 3D evaluation methods and more homogeneous endodontic sample materials so that the results offer a more consistent understanding of instrument performance and its effects on the internal anatomy of the root canal system.

This literature review has identified important factors to consider when selecting evaluation methods to evaluate rotary endodontic files; these factors are critical, not only for assessing methods selection depending on the research question, but for informing future study design to promote the harmonization of research design going forward. The factors to consider include endodontic sample type (extracted tooth versus simulated block), parameters being assessed (e.g., canal transportation versus centering ability), and the pros and cons of each evaluation method. A possible extension of this work is that future reviews could be carried out on an individual parameter to test evaluation methods by specific parameters rather than as a whole since different parameters may be optimized by different evaluation methods. In addition, a systematic review would be advised for obtaining evidence-based recommendations for establishing formal clinical guidelines for evaluation methods testing.

## Figures and Tables

**Table 1:** Studies included in reverse chronological order.

No.	Year	Author/Reference	Endodontic Sample Type(s)	Evaluation Method	Evaluation Parameters
1	2024	Shaimaa S El-Desouky [11]	Upper primary anterior teeth	CBCT	Canal transportation and centering ability
2	2024	S Swathi [12]	Premolars	High-Precision Nano-CT	Canal centering
3	2024	Bollineni A Swetha [13]	Mandibular molars	DSLR Camera	Centering capability
4	2024	Qi Zhu [14]	Maxillary first molars	MCT	Canal transportation and centering ability
5	2023	Anbarasu Subramanian [15]	Mandibular first molars	CBCT	Canal transportation and centering ability
6	2023	Tanisha Singh [16]	Mandibular molars	Cone-Beam Computed Tomography	Canal transportation and centering ability
7	2023	Simar Kaur Manocha [17]	Mandibular molars	Cone-Beam Computed Tomography	Canal transportation and centering ability
8	2023	Nadine Hawi [18]	Mandibular molars	Micro-Computed Tomography	Canal transportation and centering ability
9	2022	Vincenzo Biasillo [19]	Simulated resin blocks	Double Digital Images (Photographs) and AutoCAD	Centering ability
10	2022	Wania Christina Figueiredo Dantas [20]	Mandibular molars	Micro-Computed Tomography	Canal transportation and centering ability
11	2022	Eduardo Hideki Suzuki [21]	Mandibular molars	Cone-Beam Computed Tomography	Canal transportation and centering ability
12	2022	Selvakumar Haridoss [22]	Mandibular molars	Cone-Beam Computed Tomography	Canal transportation and centering ability
13	2022	Lu Shi [23]	Simulated resin blocks	Double Digital Images (Photographs) and Adobe Photoshop	Canal transportation and centering ability
14	2021	Thamires C de Medeiros [24]	Mandibular molars	Micro-Computed Tomography	Canal transportation and centering ability
15	2021	Mohammed Mustafa [25]	Mandibular molars	Cone-Beam Computed Tomography	Canal transportation and centering ability
16	2021	Shiva Shojaeian [26]	Mandibular molars	Micro-Computed Tomography	Canal transportation and centering ratio
17	2021	Ibrahim Faisal [27]	Mandibular molars	Micro-Computed Tomography	Canal transportation and centering ability
18	2021	A S Waly [28]	Mandibular molars	Cone-Beam Computed Tomography	Canal transportation and centering ability
19	2021	Kamil Zafar [29]	Simulated resin blocks	Double Digital Images (Photographs) and Adobe Photoshop	Canal transportation and centering ability
20	2021	Hamed Karkehabadi [30]	Mandibular molars	Cone-Beam Computed Tomography	Canal transportation and centering ratio
21	2021	Maryam Kuzekanani [31]	Maxillary and Mandibular Molars	Cone-Beam Computed Tomography	Canal transportation
22	2021	María de las Nieves Pérez Morales [32]	Mandibular molars	Micro-Computed Tomography	Canal transportation and centering ability
23	2020	Swapnil Kolhe [33]	Mandibular first molars	Micro-Compute Tomography	Canal transportation and centering ability
24	2020	Christina Razcha [34]	Mandibular molars	Micro-Computed Tomography	Canal transportation and centering ability
25	2020	P. O. F. Fernandes [35]	Mandibular molars	Micro-Computed Tomography	Canal transportation
26	2020	Burçin Arıcan Öztürk [36]	Single rooted	Cone-Beam Computed Tomography	Canal transportation and centering ability
27	2020	P. H. Htun [37]	Mandibular premolars	Micro-Computed Tomography	Canal transportation
28	2020	Mariana Mena Barreto Pivoto-João [38]	Mandibular molars	Micro-Computed Tomography	Centering ability
29	2020	Franziska Haupt [39]	Mandibular molars	Micro-Computed Tomography	Canal transportation and centering ability
30	2020	Emina Kabil [40]	Maxillary molars	Micro-Computed Tomography	Canal transportation and centering ability
31	2020	Maria de las Nieves Perez Morales [41]	Maxillary premolars	Micro-Computed Tomography	Canal transportation and centering ratio
32	2019	Peet J. van der Vyver [42]	Maxillary molars	Micro-Computed Tomography	Canal transportation and centering ratio
33	2019	Zeliha Uğur Aydın [43]	Mandibular molars	Micro-Computed Tomography	Canal transportation and centering ability
34	2019	Yousif Iqbal Nathani [44]	Mandibular premolars	Cone-Beam Computed Tomography	Canal transportation and centering ability
35	2019	Keiichiro Maki [45]	Simulated resin blocks	Double Digital Images (Photographs) and Adobe Photoshop	Centering ability
36	2019	Daniel José Filizola de Oliveira [46]	Mandibular molars	Micro-Computed Tomography	Canal transportation
37	2018	Martin Vorster [47]	Mandibular molars	Micro-Computed Tomography	Canal transportation and centering ability
38	2018	M. M. Kyaw Moe [48]	Mandibular molars	Micro-Computed Tomography	Canal transportation and centering ratio
39	2018	Mohamed Medhat Kataia [49]	Simulated resin blocks	Double Digital Images (Photographs) and Adobe Photoshop	Canal transportation
40	2018	Seyed Mohsen Hasheminia [50]	Mandibular molars	Cone-Beam Computed Tomography	Canal transportation and centering ability
41	2018	Simone Staffoli [51]	Simulated blocks	Double Digital Images (Photographs) and Adobe Photoshop	Centering ability
42	2018	E. A. Saberi [52]	Mandibular molars	Cone-Beam Computed Tomography	Canal transportation
43	2018	Guohua Yuan [53]	Mandibular molars	Micro-Computed Tomography	Canal transportation
44	2018	Pedro Marks Duarte [54]	Maxillary molars	Micro-Computed Tomography	Canal transportation and centering ability
45	2018	Felipe Gonçalves Belladonna [55]	Mandibular molars	Micro-Computed Tomography	Canal transportation
46	2017	Giulia Ferrara [56]	Mandibular and maxillary molars	Double Digital Images (Radiographs) and Adobe Photoshop	Canal transportation
47	2017	Amin A. H. Alemam [57]	Simulated resin blocks	Double Digital Images (Photographs) and Image-Pro Plus	Canal transportation
48	2017	Maurizio D’Amario [58]	Mandibular molars	Double Digital Images (Radiographs) and AutoCad	Canal transportation
49	2017	Taha Özyürek [59]	Simulated resin blocks	Double Digital Images (Photographs) and AutoCad	Canal transportation
50	2017	Caroline Zanesco [60]	Maxillary molars	Micro-Computed Tomography and Digital Radiograph	Canal transportation and centering ratio
51	2017	Pier Matteo Venino [61]	Max./Mand. molars, premolars and canine	Micro-Computed Tomography	Canal transportation and centering ratio
52	2017	Lu Shi [62]	Simulated resin blocks	Double Digital Images (Photographs) and Adobe Photoshop	Centering ability
53	2016	Ana Grasiela da Silva Limoeiro [63]	Mandibular molars	Micro-Computed Tomography	Canal transportation and centering ability
54	2016	Zhaohui Liu [64]	Premolars	Micro-Computed Tomography	Canal transportation
55	2016	Farzana Paleker [65]	Mandibular molars	Micro-Computed Tomography	Canal transportation and centering ability
56	2016	Ranya Faraj Elemam [10]	Simulated resin blocks	Double Digital Images (Photographs) and AutoCad	Canal transportation
57	2016	Filipa Neto [66]	Simulated resin blocks	Double Digital Images (Photographs) and Adobe Photoshop	Canal transportation
58	2015	Ove A. Peters [67]	Mandibular molars	Micro-Computed Tomography	Canal transportation
59	2015	Jason Gagliardi [68]	Mandibular molars	Micro-Computed Tomography	Canal transportation and centering ability
60	2015	Emmanuel João Nogueira Leal Silva [69]	Simulated resin blocks	Double Digital Images (Photographs) and Fiji	Canal transportation
61	2015	Abdulrahman Mohammed Saleh [70]	Simulated resin blocks	Double Digital Images (Photographs) and Adobe Photoshop	Canal transportation
62	2015	Damiano Pasqualini [71]	Mandibular molars	Micro-Computed Tomography	Canal transportation and centering ability
63	2015	Guilherme Moreira de Carvalho [72]	Mandibular molars	Cone-Beam Computed Tomography	Canal transportation and centering ability
64	2014	K. K. Al-Manei [73]	Mandibular molars	Double Digital Images (Photographs) and AutoCad	Canal transportation
65	2014	Amr M. Elnaghy [74]	Mandibular molars	Cone-Beam Computed Tomography	Canal transportation and centering ability
66	2014	Matthew Thompson [75]	Simulated resin blocks	Double Digital Images (Photographs) and Adobe Photoshop	Centering ability
67	2014	Dan Zhao [76]	Mandibular molars	Micro-Computed Tomography	Canal transportation
68	2014	Young-Hye Hwang [77]	Maxillary molars	Micro-Computed Tomography	Canal transportation
69	2014	Nazarimoghadam K [78]	Simulated resin blocks	Double Digital Images (Photographs) and AutoCad	Canal transportation
70	2013	Abeer M. Marzouk [79]	Mandibular molars	Cone-Beam Computed Tomography	Canal transportation
71	2013	Mina Zarei [80]	Mandibular molars	Double Digital Images (Photographs) and Adobe Photoshop	Canal transportation and centering ability
72	2012	Jeffrey R Burroughs [81]	Simulated resin blocks	Double Digital Images (Photographs) and Adobe Photoshop	Canal transportation
73	2012	Brandon Yamamura [82]	Mandibular molars	Micro-Computed Tomography	Canal transportation and centering ability
74	2012	Cumhur Aydin [83]	Simulated resin blocks	Double Digital Images (Photographs) and Adobe Photoshop	Canal transportation
75	2012	Fernando Duran-Sindreu [84]	Mandibular molars	Double Digital Images (Radiographs) and AutoCad	Canal transportation
76	2012	Ahmed Abdel Rahman Hashem [85]	Mandibular molars	Cone-Beam Computed Tomography	Canal transportation and centering ability
77	2012	Marc García [86]	Mandibular molars	Double Digital Images (Radiographs) and AutoCad	Canal transportation
78	2012	Stern S [87]	Mandibular molars	Micro-Computed Tomography	Canal transportation and centering ratio
79	2011	Guobin Yang [88]	Mandibular molars	Micro-Computed Tomography	Canal transportation and centering ability
80	2011	Hani F. Ounsi [89]	Simulated resin blocks	Double Digital Images (Photographs) and AutoCad	Canal transportation
81	2011	Laila Gonzales Freire [90]	Mandibular molars	Micro-Computed Tomography	canal transportation and centering ability
82	2011	Vittorio Franco [91]	Simulated resin blocks	Double Digital Images (Photographs) and Adobe Photoshop	Canal transportation
83	2010	Frank C. Setzer [92]	Mandibular molars	Double Digital Images (Radiographs) and AutoCad	Canal transportation
84	2010	Bekir Karabucak [93]	Mandibular molars	Double Digital Images (Radiographs) and AutoCad	Canal transportation
85	2010	Rui Gonçalves Madureira [94]	Simulated resin blocks	Double Digital Images (Radiographs) and Adobe Photoshop	Canal transportation
86	2010	Richard Gergi [95]	Mandibular molars	Cone-Beam Computed Tomography	Canal transportation and centering ratio
87	2010	Mian K. Iqbal [9]	Mandibular molars	Double Digital Images (Radiographs) and AutoCad	Canal transportation

**Table 2:** Distributions of studies per endodontic sample type, evaluation methods, and evaluation parameters.

Parameters: Endodontic Sample Type Evaluation Method	Canal Transportation	Canal Transportation and Centering Ability	Canal Transportation and Centering Ratio	Centering Ability	Total
Mandibular and maxillary molars	2				2
Cone-Beam Computed Tomography	1				1
Double Digital Images (Radiographs) and Adobe Photoshop	1				1
Mandibular molars	16	30	4	1	51
Cone-Beam Computed Tomography	2	12	2		16
Double Digital Images (Photographs) and Adobe Photoshop		1			1
Double Digital Images (Photographs) and AutoCad	1				1
Double Digital Images (Radiographs) and AutoCad	6				6
Micro-Computed Tomography	6	18	2	1	27
Digital single-lens reflex camera	1				1
Mandibular premolars	1	1			2
Cone-Beam Computed Tomography		1			1
Micro-Computed Tomography	1				1
Max./Mand. molars, premolars and canine			1		1
Micro-Computed Tomography			1		1
Maxillary molars	1	3	2		6
Micro-Computed Tomography	1	2	1		4
Micro-Computed Tomography and Digital Radiograph			1		1
Cone-Beam Computed Tomography		1			1
Maxillary premolars			1		1
Micro-Computed Tomography			1		1
Premolars	2				2
Micro-Computed Tomography	1				1
High-Precision Nano-CT	1				1
Upper primary anterior teeth		1			1
Cone-Beam Computed Tomography		1			1
Simulated blocks				1	1
Double Digital Images (Photographs) and Adobe Photoshop				1	1
Simulated resin blocks	13	2		4	19
Double Digital Images (Photographs) and Adobe Photoshop	6	2		3	11
Double Digital Images (Photographs) and AutoCad	3			1	4
Double Digital Images (Photographs) and Fiji	1				1
Double Digital Images (Photographs) and Image-Pro Plus	1				1
Double Digital Images (Photographs), Matlab, and Adobe Photoshop	1				1
Double Digital Images (Radiographs) and Adobe Photoshop	1				1
Single rooted		1			1
Cone-Beam Computed Tomography		1			1
TOTAL	35	39	7	6	87

## Data Availability

The original contributions presented in the study are included in the article; further inquiries can be directed to the corresponding author.

## References

[1] Schäfer E., Bürklein S. (2012). Impact of nickel-titanium instrumentation of the root canal on clinical outcomes: A focused review. Odontology.

[2] Hülsmann M., Peters O.A., Dummer P.M.H. (2005). Mechanical preparation of root canals: Shaping goals, techniques and means. Endod. Top..

[3] Schilder H. (1974). Cleaning and shaping the root canal. Dent. Clin. N. Am..

[4] Pansheriya E., Goel M., Gupta K.D., Ahuja R., Kaur R.D., Garg V. (2018). Comparative Evaluation of Apical Transportation and Canal Centric Ability in Apical Region of Newer nickel-titanium File Systems Using cone-beam computed tomography on Extracted Molars: An In Vitro Study. Contemp. Clin. Dent..

[5] Short J.A., Morgan L.A., Baumgartner J.C. (1997). A comparison of canal centering ability of four instrumentation techniques. J. Endod..

[6] American Association of Endodontists Glossary of Endodontic Chicago. https://www.aae.org/specialty/clinical-resources/glossary-endodontic-terms/.

[7] Kandaswamy D., Venkateshbabu N., Porkodi I., Pradeep G. (2009). Canal-centering ability: An endodontic challenge. J. Conserv. Dent..

[8] Jain A., Gupta A., Agrawal R. (2018). Comparative analysis of canal-centering ratio, apical transportation, and remaining dentin thickness between single-file systems, i.e., OneShape and WaveOne reciprocation: An in vitro study. J. Conserv. Dent..

[9] Iqbal M.K., Floratos S., Hsu Y.K., Karabucak B. (2010). An In Vitro Comparison of Profile GT and GTX Nickel-Titanium Rotary Instruments in Apical Transportation and Length Control in Mandibular Molar. J. Endod..

[10] Elemam R.F., Capelas J.A., Vaz M., Viriato N., de Lurdes Ferreira Lobo Pereira M., Azevedo Á. (2016). In vitro evaluation of root canal transportation after use of BT-Race files. Rev. Port. Estomatol. Med. Dent. Cir. Maxilofac..

[11] El-Desouky S.S., El Fahl B.N., Kabbash I.A., Hadwa S.M. (2024). Cone-beam computed tomography evaluation of shaping ability of kedo-S square and fanta AF baby rotary files compared to manual K-files in root canal preparation of primary anterior teeth. Clin. Oral Investig..

[12] Swathi S., Antony D.P., Solete P., Jeevanandan G., Vishwanathaiah S., Maganur P.C. (2024). Comparative evaluation of remaining dentin thickness, canal centering ability and apical deformity between ProFit S3 and Protaper gold—A nano CT study. Saudi Dent. J..

[13] Swetha B., Malini D.L., Burla D., Ismail P.M.S., Dahiya S., Bhasin R., Mohammed A. (2024). An In Vitro Comparative Assessment of Shaping Capacity of a Single-File System Over Multiple-File System in Root Canals. J. Pharm. Bioallied Sci..

[14] Zhu Q., Liu C., Bai B., Pei F., Tang Y., Song W., Chen X., Gu Y. (2024). Micro-computed tomographic evaluation of the shaping ability of three nickel-titanium rotary systems in the middle mesial canal of mandibular first molars: An ex vivo study based on 3D printed tooth replicas. BMC Oral Health.

[15] Subramanian A., Balasubramanian R., Jayakumar S., Harikrishnan S., Chandrasekaran R. (2023). Evaluation of Canal-centering Ability and Apical Transportation of Hyflex-EDM, OneShape, WaveOne Gold, and Reciproc Files: An Ex Vivo Study. J. Contemp. Dent. Pract..

[16] Singh T., Kumari M., Kochhar R. (2023). Comparative evaluation of canal transportation and centering ability of rotary and reciprocating file systems using cone-beam computed tomography: An in vitro study. J. Conserv. Dent..

[17] Manocha S.K., Saha S.G., Agarwal R.S., Vijaywargiya N., Saha M.K., Surana A. (2023). Comparative evaluation of canal transportation and canal centering ability in oval canals with newer nickel-titanium rotary single file systems—A cone-beam computed tomography study. J. Conserv. Dent..

[18] Hawi N., Pedulla E., La Rosa G.R.M., Conte G., Nehme W., Neelakantan P. (2023). Influence of Coronal Flaring on the Shaping Ability of Two Heat-Treated Nickel-Titanium Endodontic Files: A Micro-Computed Tomographic Study. J. Clin. Med..

[19] Biasillo V., Castagnola R., Colangeli M., Panzetta C., Minciacchi I., Plotino G., Staffoli S., Marigo L., Grande N.M. (2022). Comparison of shaping ability of the Reciproc Blue and One Curve with or without glide path in simulated S-shaped root canals. Restor. Dent. Endod..

[20] Dantas W.C.F., Marceliano-Alves M.F.V., Marceliano E.F.V., Marques E.F., de Carvalho Coutinho T.M., Alves F.R.F., Martin A.S., Pelegrine R.A., Lopes R.T., Bueno C. (2023). Microtomographic Assessment of the Shaping Ability of the Hyflex CM and XP-endo Shaper Systems in Curved Root Canals. Eur. J. Dent..

[21] Suzuki E.H., Sponchiado-Junior E.C., Pandolfo M.T., Garcia L., Carvalho F.M.A., Marques A.A.F. (2022). Shaping Ability of Reciprocating and Rotary Systems After Root Canal Retreatment: A CBCT Study. Braz. Dent. J..

[22] Haridoss S., Rakkesh K.M., Swaminathan K. (2022). Transportation and Centering Ability of Kedo-S Pediatric and Mtwo Instruments in Primary Teeth: A Cone-beam ComputedTomography Study. Int. J. Clin. Pediatr. Dent..

[23] Shi L., Zhou J., Wan J., Yang Y. (2022). Shaping ability of ProTaper Gold and WaveOne Gold nickel-titanium rotary instruments in simulated S-shaped root canals. J. Dent. Sci..

[24] Medeiros T.C., Lima C.O., Barbosa A.F.A., Augusto C.M., Bruno A.M.V., Lopes R.T., Amoroso-Silva P.A., Marceliano-Alves M.F. (2021). Shaping ability of reciprocating and rotary systems in oval-shaped root canals: A microcomputed tomography study. Acta Odontol. Latinoam..

[25] Mustafa M. (2021). Comparative Evaluation of Canal-shaping Abilities of RaceEvo, R-Motion, Reciproc Blue, and ProTaper Gold NiTi Rotary File Systems: A CBCT Study. J. Contemp. Dent. Pract..

[26] Shojaeian S., Mortezapour N., Soltaninejad F., Zargar N., Zandi B., Shantiaee Y., Bidaki A. (2021). Comparison of Canal Transportation and Centering Ability of One-G, EdgeGlidePath, and Neolix: A MicroComputed Tomography Study of Curved Root Canals. Int. J. Dent..

[27] Faisal I., Saif R., Alsulaiman M., Natto Z.S. (2021). Shaping ability of 2Shape and NeoNiTi rotary instruments in preparation of curved canals using micro-computed tomography. BMC Oral Health.

[28] Waly A.S., Yamany I., Abbas H.M., MA A.A., RM F.B., Bogari D.F., Alhazzazi T.Y. (2021). Comparison of two pediatric rotary file systems and hand instrumentation in primary molar: An ex vivo cone-beam computed tomographic study. Niger. J. Clin. Pract..

[29] Zafar K., Badar S.B., Ghafoor R., Khan F.R. (2021). Comparison Of Centering Ability And Transportation Of The Protaper Next And Oneshape File Rotary Systems For Preparing Simulated Curved Canal. J. Ayub. Med. Coll. Abbottabad..

[30] Karkehabadi H., Siahvashi Z., Shokri A., Haji Hasani N. (2021). Cone-beam computed tomographic analysis of apical transportation and centering ratio of ProTaper and XP-endo Shaper NiTi rotary systems in curved canals: An in vitro study. BMC Oral Health.

[31] Kuzekanani M., Sadeghi F., Hatami N., Rad M., Darijani M., Walsh L.J. (2021). Comparison of Canal Transportation, Separation Rate, and Preparation Time between One Shape and Neoniti (Neolix): An In Vitro CBCT Study. Int. J. Dent..

[32] Pérez Morales M.d.l.N., González Sánchez J.A., Olivieri J.G., Elmsmari F., Salmon P., Jaramillo D.E., Duran-Sindreu F. (2021). Micro-computed Tomographic Assessment and Comparative Study of the Shaping Ability in Six NiTi files—An In Vitro Study. J. Endod..

[33] Kolhe S.J., Kolhe P.S., Gulve M.N., Aher G.B., Bhadage C.J., Mashalkar S.S. (2020). Microcomputed tomographic evaluation of shaping ability of two thermo mechanically treated single-file systems in severely curved roots. J. Conserv. Dent..

[34] Razcha C., Zacharopoulos A., Anestis D., Mikrogeorgis G., Zacharakis G., Lyroudia K. (2020). Micro-Computed Tomographic Evaluation of Canal Transportation and Centering Ability of 4 Heat-Treated Nickel-Titanium Systems. J. Endod..

[35] Fernandes P.O.F., Freire L.G., Iglecias E.F., Vieira B.R., Zuolo M.L., Gavini G. (2020). Assessment of Mechanical Root Canal Preparation with Centric Reciprocating or Eccentric Rotary Kinematics: A Micro–computed Tomographic Study. J. Endod..

[36] Arıcan Öztürk B., Atav Ateş A., Fişekçioğlu E. (2020). Cone-Beam Computed Tomographic Analysis of Shaping Ability of XP-endo Shaper and ProTaper Next in Large Root Canals. J. Endod..

[37] Htun P.H., Ebihara A., Maki K., Kimura S., Nishijo M., Okiji T. (2020). Cleaning and Shaping Ability of Gentlefile, HyFlex EDM, and ProTaper Next Instruments: A Combined Micro–computed Tomographic and Scanning Electron Microscopic Study. J. Endod..

[38] Pivoto-João M.M.B., Tanomaru-Filho M., Pinto J.C., Espir C.G., Guerreiro-Tanomaru J.M. (2020). Root Canal Preparation and Enlargement Using Thermally Treated Nickel-Titanium Rotary Systems in Curved Canals. J. Endod..

[39] Haupt F., Pult J.R.W., Hülsmann M. (2020). Micro–computed Tomographic Evaluation of the Shaping Ability of 3 Reciprocating Single-File Nickel-Titanium Systems on Single- and Double-Curved Root Canals. J. Endod..

[40] Kabil E., Katić M., Anić I., Bago I. (2020). Micro–computed Evaluation of Canal Transportation and Centering Ability of 5 Rotary and Reciprocating Systems with Different Metallurgical Properties and Surface Treatments in Curved Root Canals. J. Endod..

[41] Perez Morales M.d.l.N., González Sánchez J.A., Olivieri Fernández J.G., Laperre K., Abella Sans F., Jaramillo D.E., Terol F.D.-S. (2020). TRUShape Versus XP-endo Shaper: A Micro–computed Tomographic Assessment and Comparative Study of the Shaping Ability—An In Vitro Study. J. Endod..

[42] van der Vyver P.J., Paleker F., Vorster M., de Wet F.A. (2019). Root Canal Shaping Using Nickel Titanium, M-Wire, and Gold Wire: A Micro–computed Tomographic Comparative Study of One Shape, ProTaper Next, and WaveOne Gold Instruments in Maxillary First Molars. J. Endod..

[43] Aydın Z.U., Keskin N.B., Özyürek T., Geneci F., Ocak M., Çelik H.H. (2019). Microcomputed Assessment of Transportation, Centering Ratio, Canal Area, and Volume Increase after Single-file Rotary and Reciprocating Glide Path Instrumentation in Curved Root Canals: A Laboratory Study. J. Endod..

[44] Nathani T.I., Nathani A.I., Pawar A.M., Khakiani M.I., Ruiz X.-F., Olivieri J.G. (2019). Canal Transportation and Centering Ability in Long Oval Canals: A Multidimentional Analysis. J. Endod..

[45] Maki K., Ebihara A., Kimura S., Nishijo M., Tokita D., Okiji T. (2019). Effect of Different Speeds of Up-and-down Motion on Canal Centering Ability and Vertical Force and Torque Generation of Nickel-titanium Rotary Instruments. J. Endod..

[46] Filizola de Oliveira D.J., Leoni G.B., da Silva Goulart R., Sousa-Neto M.D.d., Silva Sousa Y.T.C., Silva R.G. (2019). Changes in Geometry and Transportation of Root Canals with Severe Curvature Prepared by Different Heat-treated Nickel-titanium Instruments: A Micro–computed Tomographic Study. J. Endod..

[47] Vorster M., van der Vyver P.J., Paleker F. (2018). Canal Transportation and Centering Ability of WaveOne Gold in Combination with and without Different Glide Path Techniques. J. Endod..

[48] Kyaw Moe M.M., Ha J.H., Jin M.U., Kim Y.K., Kim S.K. (2018). Root Canal Shaping Effect of Instruments with Offset Mass of Rotation in the Mandibular First Molar: A Micro–computed Tomographic Study. J. Endod..

[49] Kataia M.M., Roshdy N.N., Nagy M.M. (2018). Comparative analysis of canal transportation using reciproc blue and wavo one gold in simulated root canals using different kinematics. Future Dent. J..

[50] Hasheminia S.M., Farhad A., Sheikhi M., Soltani P., Hendi S.S., Ahmadi M. (2018). Cone-beam Computed Tomographic Analysis of Canal Transportation and Centering Ability of Single-file Systems. J. Endod..

[51] Staffoli S., Özyürek T., Hadad A., Lvovsky A., Solomonov M., Azizi H., Itzhak J.B., Bossù M., Grande N.M., Plotino G. (2018). Comparison of shaping ability of ProTaper Next and 2Shape nickel–titanium files in simulated severe curved canals. Giornale Ital. Endod..

[52] Saberi E.A., Mollashahi N.F., Farahi F. (2018). Canal transportation caused by one single-file and two multiple-file rotary systems: A comparative study using cone-beam computed tomography. Giornale Ital. Endod..

[53] Yuan G., Yang G. (2018). Comparative evaluation of the shaping ability of single-file system versus multi-file system in severely curved root canals. J. Dent. Sci..

[54] Marks Duarte P., Barcellos da Silva P., Alcalde M.P., Vivan R.R., Rosa R.A.d., Duarte M.A.H., Só M.V.R. (2018). Canal Transportation, Centering Ability, and Cyclic Fatigue Promoted by Twisted File Adaptive and Navigator EVO Instruments at Different Motions. J. Endod..

[55] Belladonna F.G., Carvalho M.S., Cavalcante D.M., Fernandes J.T., de Carvalho Maciel A.C., Oliveira H.E., Lopes R.T., Silva E.J.N.L., De-Deus G. (2018). Micro–computed Tomography Shaping Ability Assessment of the New Blue Thermal Treated Reciproc Instrument. J. Endod..

[56] Ferrara G., Taschieri S., Corbella S., Ceci C., Del Fabbro M., Machtou P. (2017). Comparative evaluation of the shaping ability of two different nickel-titanium rotary files in curved root canals of extracted human molar teeth. J. Investig. Clin. Dent..

[57] Alemam A.A.H., Dummer P.M.H., Farnell D.J.J. (2017). A Comparative Study of ProTaper Universal and ProTaper Next Used by Undergraduate Students to Prepare Root Canals. J. Endod..

[58] D’Amario M., De Angelis F., Mancino M., Frascaria M., Capogreco M., D’Arcangelo C. (2017). Canal shaping of different single-file systems in curved root canals. J. Dent. Sci..

[59] Özyürek T., Yılmaz K., Uslu G. (2017). Shaping Ability of Reciproc, WaveOne GOLD, and HyFlex EDM Single-file Systems in Simulated S-shaped Canals. J. Endod..

[60] Zanesco C., Só M.V.R., Schmidt S., Fontanella V.R.C., Grazziotin-Soares R., Barletta F.B. (2017). Apical Transportation, Centering Ratio, and Volume Increase after Manual, Rotary, and Reciprocating Instrumentation in Curved Root Canals: Analysis by Micro-computed Tomographic and Digital Subtraction Radiography. J. Endod..

[61] Venino P.M., Citterio C.L., Pellegatta A., Ciccarelli M., Maddalone M. (2017). A Micro–computed Tomography Evaluation of the Shaping Ability of Two Nickel-titanium Instruments, HyFlex EDM and ProTaper Next. J. Endod..

[62] Shi L., Wagle S. (2017). Comparing the Centering Ability of Different Pathfinding Systems and Their Effect on Final Instrumentation by Hyflex CM. J. Endod..

[63] da Silva Limoeiro A.G., dos Santos A.H.B., De Martin A.S., Kato A.S., Fontana C.E., Gavini G., Freire L.G., da Silveira Bueno C.E. (2016). Micro-Computed Tomographic Evaluation of 2 Nickel-Titanium Instrument Systems in Shaping Root Canals. J. Endod..

[64] Liu Z., Liu J., Gu L., Liu W., Wei X., Ling J. (2016). The shaping and cleaning abilities of self-adjusting files in the preparation of canals with isthmuses after glidepath enlargement with ISO or ProTaper Universal NiTi files. J. Dent. Sci..

[65] Paleker F., van der Vyver P.J. (2016). Comparison of Canal Transportation and Centering Ability of K-files, ProGlider File, and G-Files: A Micro-Computed Tomography Study of Curved Root Canals. J. Endod..

[66] Neto F., Ginjeira A. (2016). Comparative analysis of simulated root canals shaping, using ProTaper Universal, Next and Gold. Rev. Port. Estomatol. Med. Dent. Cir. Maxilofac..

[67] Peters O.A., Arias A., Paqué F. (2015). A Micro–computed Tomographic Assessment of Root Canal Preparation with a Novel Instrument, TRUShape, in Mesial Roots of Mandibular Molars. J. Endod..

[68] Gagliardi J., Versiani M.A., de Sousa-Neto M.D., Plazas-Garzon A., Basrani B. (2015). Evaluation of the Shaping Characteristics of ProTaper Gold, ProTaper NEXT, and ProTaper Universal in Curved Canals. J. Endod..

[69] Silva E.J.N.L., Tameirão M.D.N., Belladonna F.G., Neves A.A., Souza E.M., De-Deus G. (2015). Quantitative Transportation Assessment in Simulated Curved Canals Prepared with an Adaptive Movement System. J. Endod..

[70] Saleh A.M., Vakili Gilani P., Tavanafar S., Schäfer E. (2015). Shaping Ability of 4 Different Single-file Systems in Simulated S-shaped Canals. J. Endod..

[71] Pasqualini D., Alovisi M., Cemenasco A., Mancini L., Paolino D.S., Bianchi C.C., Roggia A., Scotti N., Berutti E. (2015). Micro–Computed Tomography Evaluation of ProTaper Next and BioRace Shaping Outcomes in Maxillary First Molar Curved Canals. J. Endod..

[72] de Carvalho G.M., Sponchiado Junior E.C., Garrido A.D.B., Lia R.C.C., Roberti Garcia L.d.F., Franco Marques A.A. (2015). Apical Transportation, Centering Ability, and Cleaning Effectiveness of Reciprocating Single-file System Associated with Different Glide Path Techniques. J. Endod..

[73] Al-Manei K.K., Al-Hadlaq S.M.S. (2014). Evaluation of the root canal shaping ability of two rotary nickel–titanium systems. Int. Endod. J..

[74] Elnaghy A.M., Elsaka S.E. (2014). Evaluation of root canal transportation, centering ratio, and remaining dentin thickness associated with ProTaper Next instruments with and without glide path. J. Endod..

[75] Thompson M., Sidow S.J., Lindsey K., Chuang A., McPherson J.C. (2014). Evaluation of a New Filing System’s Ability to Maintain Canal Morphology. J. Endod..

[76] Zhao D., Shen Y., Peng B., Haapasalo M. (2014). Root Canal Preparation of Mandibular Molars with 3 Nickel-Titanium Rotary Instruments: A Micro–Computed Tomographic Study. J. Endod..

[77] Hwang Y.-H., Bae K.-S., Baek S.-H., Kum K.-Y., Lee W., Shon W.-J., Chang S.W. (2014). Shaping Ability of the Conventional Nickel-Titanium and Reciprocating Nickel-Titanium File Systems: A Comparative Study Using Micro–Computed Tomography. J. Endod..

[78] Nazarimoghadam K., Daryaeian M., Ramazani N. (2014). An in vitro comparison of root canal transportation by reciproc file with and without glide path. J. Dent..

[79] Marzouk A.M., Ghoneim A.G. (2013). Computed Tomographic Evaluation of Canal Shape Instrumented by Different Kinematics Rotary Nickel-Titanium Systems. J. Endod..

[80] Zarei M., Javidi M., Erfanian M., Lomee M., Afkhami F. (2013). Comparison of air-driven vs electric torque control motors on canal centering ability by ProTaper NiTi rotary instruments. J. Contemp. Dent. Pract..

[81] Burroughs J.R., Bergeron B.E., Roberts M.D., Hagan J.L., Himel V.T. (2012). Shaping ability of three nickel-titanium endodontic file systems in simulated S-shaped root canals. J. Endod..

[82] Yamamura B., Cox T.C., Heddaya B., Flake N.M., Johnson J.D., Paranjpe A. (2012). Comparing Canal Transportation and Centering Ability of EndoSequence and Vortex Rotary Files by Using Micro–Computed Tomography. J. Endod..

[83] Aydin C., Inan U., Gultekin M. (2012). Comparison of the shaping ability of Twisted Files with ProTaper and RevoS nickel-titanium instruments in simulated canals. J. Dent. Sci..

[84] Duran-Sindreu F., García M., Olivieri J.G., Mercadé M., Morelló S., Roig M. (2012). A Comparison of Apical Transportation between FlexMaster and Twisted Files Rotary Instruments. J. Endod..

[85] Hashem A.A.R., Ghoneim A.G., Lutfy R.A., Foda M.Y., Omar G.A.F. (2012). Geometric Analysis of Root Canals Prepared by Four Rotary NiTi Shaping Systems. J. Endod..

[86] García M., Duran-Sindreu F., Mercadé M., Bueno R., Roig M. (2012). A Comparison of Apical Transportation between ProFile and RaCe Rotary Instruments. J. Endod..

[87] Stern S., Patel S., Foschi F., Sherriff M., Mannocci F. (2012). Changes in centring and shaping ability using three nickel–titanium instrumentation techniques analysed by micro-computed tomography (μCT). Int. Endod. J..

[88] Yang G., Yuan G., Yun X., Zhou X., Liu B., Wu H. (2011). Effects of Two Nickel-Titanium Instrument Systems, Mtwo versus ProTaper Universal, on Root Canal Geometry Assessed by Micro–Computed Tomography. J. Endod..

[89] Ounsi H.F., Franciosi G., Paragliola R., Al Huzaimi K., Salameh Z., Tay F.R., Ferrari M., Grandini S. (2011). Comparison of Two Techniques for Assessing the Shaping Efficacy of Repeatedly Used Nickel-Titanium Rotary Instruments. J. Endod..

[90] Freire L.G., Gavini G., Branco-Barletta F., Sanches-Cunha R., dos Santos M. (2011). Microscopic computerized tomographic evaluation of root canal transportation prepared with twisted or ground nickel-titanium rotary instruments. Oral Surg. Oral Med. Oral Pathol. Oral Radiol. Endodontol..

[91] Franco V., Fabiani C., Taschieri S., Malentacca A., Bortolin M., Del Fabbro M. (2011). Investigation on the Shaping Ability of Nickel-Titanium Files When Used with a Reciprocating Motion. J. Endod..

[92] Setzer F.C., Kwon T.-K., Karabucak B. (2010). Comparison of Apical Transportation between Two Rotary File Systems and Two Hybrid Rotary Instrumentation Sequences. J. Endod..

[93] Karabucak B., Gatan A.J., Hsiao C., Iqbal M.K. (2010). A Comparison of Apical Transportation and Length Control between EndoSequence and Guidance Rotary Instruments. J. Endod..

[94] Madureira R.G., Forner Navarro L., Llena M.C., Costa M. (2010). Shaping ability of nickel-titanium rotary instruments in simulated S-shaped root canals. Oral Surg. Oral Med. Oral Pathol. Oral Radiol. Endodontol..

[95] Gergi R., Rjeily J.A., Sader J., Naaman A. (2010). Comparison of Canal Transportation and Centering Ability of Twisted Files, Pathfile-ProTaper System, and Stainless Steel Hand K-Files by Using Computed Tomography. J. Endod..

[96] Yin X., Cheung G.S.-P., Zhang C., Masuda Y.M., Kimura Y., Matsumoto K. (2010). Micro-computed Tomographic Comparison of Nickel-Titanium Rotary versus Traditional Instruments in C-Shaped Root Canal System. J. Endod..

[97] Cai H.X., Cheng H.L., Song J.W., Chen S.Y. (2014). Comparison of Hero 642 and K3 rotary nickel-titanium files in curved canals of molars and a systematic review of the literature. Exp. Ther. Med..

[98] Kirkevang L.L., Hörsted-Bindslev P., Orstavik D., Wenzel A. (2001). A comparison of the quality of root canal treatment in two Danish subpopulations examined 1974-75 and 1997-98. Int. Endod. J..

[99] Bjørndal L., Laustsen M.H., Reit C. (2006). Root canal treatment in Denmark is most often carried out in carious vital molar teeth and retreatments are rare. Int. Endod. J..

[100] de Oliveira Alves V., da Silveira Bueno C.E., Cunha R.S., Pinheiro S.L., Fontana C.E., de Martin A.S. (2012). Comparison among Manual Instruments and PathFile and Mtwo Rotary Instruments to Create a Glide Path in the Root Canal Preparation of Curved Canals. J. Endod..

[101] Berutti E., Paolino D.S., Chiandussi G., Alovisi M., Cantatore G., Castellucci A., Pasqualini D. (2012). Root canal anatomy preservation of WaveOne reciprocating files with or without glide path. J. Endod..

[102] Gambill J.M., Alder M., del Rio C.E. (1996). Comparison of nickel-titanium and stainless steel hand-file instrumentation using computed tomography. J. Endod..

[103] Rödig T., Hülsmann M., Mühge M., Schäfers F. (2002). Quality of preparation of oval distal root canals in mandibular molars using nickel-titanium instruments. Int. Endod. J..

[104] Iqbal M.K., Maggiore F., Suh B., Edwards K.R., Kang J., Kim S. (2003). Comparison of Apical Transportation in Four Ni-Ti Rotary Instrumentation Techniques. J. Endod..

[105] Javaheri H.H., Javaheri G.H. (2007). A Comparison of Three Ni-Ti Rotary Instruments in Apical Transportation. J. Endod..

[106] dos Santos M.D.B., Marceliano M.F., Silva E Souza P.R.d.A. (2006). Evaluation of apical deviation in root canals instrumented with K3 and ProTaper systems. J. Appl. Oral Sci. Rev. FOB.

[107] Mouyen F., Benz C., Sonnabend E., Lodter J.P. (1989). Presentation and physical evaluation of RadioVisioGraphy. Oral Surg. Oral Med. Oral Pathol..

[108] Abou-Rass M., Frank A.L., Glick D.H. (1980). The anticurvature filing method to prepare the curved root canal. J. Am. Dent. Assoc..

[109] Bramante C.M., Berbert A., Borges R.P. (1987). A methodology for evaluation of root canal instrumentation. J. Endod..

[110] Dowker S.E.P., Davis G.R., Elliott J.C. (1997). X-ray microtomography: Nondestructive three-dimensional imaging for in vitro endodontic studies. Oral Surg. Oral Med. Oral Pathol. Oral Radiol. Endodontol..

[111] Backman C.A., Oswald R.J., Pitts D.L. (1992). A radiographic comparison of two root canal instrumentation techniques. J. Endod..

[112] Hülsmann M., Stryga F. (1993). Comparison of root canal preparation using different automated devices and hand instrumentation. J. Endod..

[113] Ounsi H.F., Franciosi G., Paragliola R., Goracci C., Grandini S. (2010). Effect of repeated use on the shaping ability of Protaper Universal rotary files. Int. Dent. SA.

[114] Maggiore F. (1993–1994). Endodontic Preparation of Curved Root Canals Using the Mac Files: Evaluation Using a Radiographic Method and a Computerized Analysis. Master’s Thesis.

[115] Bahia M.G., Buono V.T. (2005). Decrease in the fatigue resistance of nickel-titanium rotary instruments after clinical use in curved root canals. Oral Surg. Oral Med. Oral Pathol. Oral Radiol. Endod..

[116] Marceliano-Alves M.F.V., Sousa-Neto M.D., Fidel S.R., Steier L., Robinson J.P., Pécora J.D., Versiani M.A. (2015). Shaping ability of single-file reciprocating and heat-treated multifile rotary systems: A micro-CT study. Int. Endod. J..

[117] Moore J., Fitz-Walter P., Parashos P. (2009). A micro-computed tomographic evaluation of apical root canal preparation using three instrumentation techniques. Int. Endod. J..

[118] Dufresne T., Chmielewski P., Borah B., Laib A. (2004). Microcomputed tomography and its applications. Encyclopaedia of Biomaterials and Biomedical Engineering.

[119] Marciano M., Duarte M., Ordinola-Zapata R., Del Carpio-Perochena A., Cavenago B., Villas Bôas M., Minotti P.G., Bramante C., Moraes I.G. (2012). Applications of micro-computed tomography in endodontic research. Curr. Microsc. Contrib. Adv. Sci. Technol..

[120] Liang X., Zhang Z., Gu J., Wang Z., Vandenberghe B., Jacobs R., Yang J., Ma G., Ling H., Ma X. (2017). Comparison of micro-CT and cone beam CT on the feasibility of assessing trabecular structures in mandibular condyle. Dentomaxillofac. Radiol..

[121] Borna Z., Rahimi S., Shahi S., Zand V. (2011). Mandibular second premolars with three root canals: A review and 3 case reports. Iran. Endod. J..

[122] Fan B., Pan Y., Gao Y., Fang F., Wu Q., Gutmann J.L. (2010). Three-dimensional morphologic analysis of isthmuses in the mesial roots of mandibular molars. J. Endod..

[123] Peters O.A., Paqué F. (2011). Root canal preparation of maxillary molars with the self-adjusting file: A micro-computed tomography study. J. Endod..

[124] Nielsen R.B., Alyassin A.M., Peters D.D., Carnes D.L., Lancaster J. (1995). Microcomputed tomography: An advanced system for detailed endodontic research. J. Endod..

[125] Peters O.A., Laib A., Göhring T.N., Barbakow F. (2001). Changes in root canal geometry after preparation assessed by high-resolution computed tomography. J. Endod..

[126] Stavileci M., Hoxha V., Görduysus Ö., Tatar I., Laperre K., Hostens J., Küçükkaya S., Berisha M. (2013). Effects of preparation techniques on root canal shaping assessed by micro-computed tomography. Med. Sci. Monit. Basic Res..

[127] Gundappa M., Bansal R., Khoriya S., Mohan R. (2014). Root canal centering ability of rotary cutting nickel titanium instruments: A meta-analysis. J. Conserv. Dent..

[128] Peyrin F., Dong P., Pacureanu A., Langer M. (2014). Micro- and nano-CT for the study of bone ultrastructure. Curr. Osteoporos. Rep..

[129] Ahmed H.M. (2016). Nano-computed tomography: Current and future perspectives. Restor. Dent. Endod..

[130] Saber S.E.D.M., El Sadat S.M.A. (2013). Effect of Altering the Reciprocation Range on the Fatigue Life and the Shaping Ability of WaveOne Nickel-Titanium Instruments. J. Endod..

[131] Rhodes J.S., Ford T.R.P., Lynch J.A., Liepins P.J., Curtis R.V. (1999). Micro-computed tomography: A new tool for experimental endodontology. Int. Endod. J..

[132] Gluskin A.H., Brown D.C., Buchanan L.S. (2001). A reconstructed computerized tomographic comparison of Ni–Ti rotary GT™ files versus traditional instruments in canals shaped by novice operators. Int. Endod. J..

[133] Patel S., Kanagasingam S., Mannocci F. (2010). Cone beam computed tomography (CBCT) in endodontics. Dent. Update.

[134] Patel S., Dawood A., Ford T.P., Whaites E. (2007). The potential applications of cone beam computed tomography in the management of endodontic problems. Int. Endod. J..

[135] Cohenca N., Shemesh H. (2015). Clinical applications of cone beam computed tomography in endodontics: A comprehensive review. Quintessence Int..

[136] Hartmann M.S.M., Barletta F.B., Camargo Fontanella V.R., Vanni J.R. (2007). Canal Transportation after Root Canal Instrumentation: A Comparative Study with Computed Tomography. J. Endod..

[137] Dhingra A., Banerjee S., Yadav V., Aggarwal N. (2014). Canal Shaping with ProTaper Next and ProTaper Universal: A Comparative Study. Ann. Dent. Res..

[138] Jain A., Asrani H., Singhal A.C., Bhatia T.K., Sharma V., Jaiswal P. (2016). Comparative evaluation of canal transportation, centering ability, and remaining dentin thickness between WaveOne and ProTaper rotary by using cone beam computed tomography: An in vitro study. J. Conserv. Dent..

[139] Pires M., Martins J.N.R., Pereira M.R., Vasconcelos I., Costa R.P.D., Duarte I., Ginjeira A. (2024). Diagnostic value of cone beam computed tomography for root canal morphology assessment—A micro-CT based comparison. Clin. Oral Investig..

[140] Peters O.A., Schönenberger K., Laib A. (2001). Effects of four Ni-Ti preparation techniques on root canal geometry assessed by micro computed tomography. Int. Endod. J..

[141] Nemtoi A., Czink C., Haba D., Gahleitner A. (2013). Cone beam CT: A current overview of devices. Dentomaxillofac. Radiol..

[142] Oliveira C.A.P., Meurer M.I., Pascoalato C., Silva S.R.C. (2009). Cone-beam computed tomography analysis of the apical third of curved roots after mechanical preparation with different automated systems. Braz. Dent. J..

